# Molecular Characterization of a Novel Cold-Active Hormone-Sensitive Lipase (*Ha*HSL) from *Halocynthiibacter Arcticus*

**DOI:** 10.3390/biom9110704

**Published:** 2019-11-05

**Authors:** Ly Thi Huong Luu Le, Wanki Yoo, Changwoo Lee, Ying Wang, Sangeun Jeon, Kyeong Kyu Kim, Jun Hyuck Lee, T. Doohun Kim

**Affiliations:** 1 Department of Chemistry, College of Natural Science, Sookmyung Women’s University, Seoul 04310, Korea; ly.12000532@gmail.com (L.T.H.L.L.); vlqkshqk61@outlook.kr (W.Y.); yingaabb@gmail.com (Y.W.); sangeun94@sookmyung.ac.kr (S.J.); 2 Department of Molecular Cell Biology, Samsung Biomedical Research Institute, Sungkyunkwan University School of Medicine, Suwon 440-746, Korea; kyeongkyu@skku.edu; 3 Department of Polar Sciences, University of Science and Technology (UST), Incheon 21990, Korea; justay@kopri.re.kr (C.L.); junhyucklee@kopri.re.kr (J.H.L.); 4 Unit of Research for Practical Application, Korea Polar Research Institute (KOPRI), Incheon 21990, Korea

**Keywords:** hormone-sensitive lipase, substrate specificity, enantioselectivity, immobilization, *Halocynthiibacter arcticus*

## Abstract

Bacterial hormone-sensitive lipases (bHSLs), which are homologous to the catalytic domains of human HSLs, have received great interest due to their uses in the preparation of highly valuable biochemicals, such as drug intermediates or chiral building blocks. Here, a novel cold-active HSL from *Halocynthiibacter arcticus* (*Ha*HSL) was examined and its enzymatic properties were investigated using several biochemical and biophysical methods. Interestingly, *Ha*HSL acted on a large variety of substrates including tertiary alcohol esters and fish oils. Additionally, this enzyme was highly tolerant to high concentrations of salt, detergents, and glycerol. Furthermore, immobilized *Ha*HSL retained its activity for up to six cycles of use. Homology modeling suggested that aromatic amino acids (Trp^23^, Tyr^74^, Phe^78^, Trp^83^, and Phe^245^) in close proximity to the substrate-binding pocket were important for enzyme activity. Mutational analysis revealed that Tyr^74^ played an important role in substrate specificity, thermostability, and enantioselectivity. In summary, the current study provides an invaluable insight into the novel cold-active *Ha*HSL from *H. arcticus*, which can be efficiently and sustainably used in a wide range of biotechnological applications.

## 1. Introduction

Lipases (EC 3.1.1.3) are mainly involved in the formation and cleavage of ester bonds between carboxylic and hydroxyl groups in a large variety of substrates, including triacylglycerols, fatty acid esters, and carbohydrates [1,2]. These enzymes, identified across all three kingdoms, contain a functional serine at their active site. Due to their high substrate specificity and strong stereoselectivity, lipases are widely utilized in the preparation of pharmaceuticals, biopolymers, food ingredients, cosmetics, and agrochemicals [3,4]. Among the lipase families, hormone-sensitive lipases (HSLs, E.C. 3.1.1.79) in mammals are involved in the hydrolysis of triacylglycerols during lipid metabolism and energy homeostasis [5,6]. Furthermore, HSLs have been shown to have various substrates, such as cholesteryl esters, fatty acids esters, and steroids. HSLs consist of an N-terminal interaction domain and a C-terminal catalytic domain [7]. Recently, bacterial enzymes (collectively termed as bacterial HSLs), which consist of a catalytic domain that is highly homologous to their mammalian counterparts, have been identified and characterized in microorganisms [8,9]. The bacterial HSL family has the characteristic α/β hydrolase fold with the highly conserved Ser-His-Asp(Glu) catalytic triad, with the catalytic Ser present within a conserved GxSAGG motif. Additionally, an HGGG motif is present, which is involved in the formation of the oxyanion hole [10].

Due to the high demands for novel biocatalysts and enzymes from extreme habitats, a number of studies have been conducted to identify cold-active enzymes with unique properties for industrial applications [11,12,13]. In this study, we characterize and immobilize a novel hormone sensitive lipase (*Ha*HSL) from *Halocynthiibacter arcticus*, and used site-directed mutagenesis to identify key amino acids. *H. arcticus*, an aerobic, gram-negative, and rod-shaped bacteria, was previously isolated from a marine sediment from the coast of Svalbard in the Arctic (78° 55′ N 11° 53′ E) [14]. The complete genome comprised ≈4.3 M nucleotides with 4675 predicted protein-coding genes [15]. Although the genomic sequences of *H. arcticus* is currently available, information about the gene products of this psychrophilic bacterium are yet to be revealed.

Although a number of bacterial HSLs were identified and characterized, including *Salinisphaera sp.* P7-4, *Staphylococcus saprophytius* AG1, *Altererythrobacter epoxidivorans,* and *Bacillus halodurans*, there is limited information available on cold-active HSLs from psychrophilic microorganisms [8]. *H. arcticus* could be an invaluable source of novel cold-active enzymes for biotechnological applications. This study paves the way for the molecular understanding of the cold-active bacterial HSL family and describes the preparation of a potential novel industrial biocatalyst. Additionally, this is the first report characterizing a novel halotolerant enzyme from the psychrophilic *H. arcticus*.

## 2. Materials and Methods

### 2.1. Reagents

T4 DNA ligase and restriction endonucleases were obtained from New England BioLabs (Ipswich, MA, USA). DNA purification kits including plasmid purification, polymerase chain reaction (PCR) purification, and gel extraction kits were obtained from Qiagen Korea (Daejon, Korea). HisTrap HP and PD-10 protein purification columns were purchased from GE Healthcare Korea (Seoul, Korea). Preparation of racemic and (*S*)-naproxol acetate was described previously [16,17]. All other reagents were of analytical grade and were purchased from Sigma-Aldrich Korea (Yongin, Korea).

### 2.2. Cloning and Purification

*H. arcticus* (KCTC 42129, Korean Collection for Type Cultures, Seoul, Korea) were cultured in Marine Agar 2216 (BD Difco, Franklin Lakes, NJ, USA) and chromosomal DNA was purified using a DNeasy Tissue and Blood Kit (Qiagen, Germantown, MD, USA). The open reading frame of the *Ha*HSL gene was amplified using PCR from the genomic DNA of *H. arcticus*, and the PCR product was cloned into pET-21a using *NheI* and *XhoI*. The following primers were used (forward primer: 5′-GATTT GCTAGC ATGGACATGGACGACG-3′, and reverse primer: 5′-GCATT CTCGAG GATAAGG GCTTTCATC-3′). After confirming the DNA sequence, the recombinant plasmid (pET-*Ha*HSL) was used for *Ha*HSL protein expression. *Escherichia coli* BL21(λDE3) cells were grown in LB medium until the optical density (OD_600nm_) reached ≈0.5–0.6. After induction with 1 mM isopropyl-β-D-1-thiogalactoside for 4 h at 37 °C, bacterial cells were harvested via centrifugation at 3000× g for 20 min at 4 °C, and then re-suspended in a 50 mL lysis buffer (20 mM Tris-HCl pH 7.4, 300 mM NaCl, 20 mM imidazole, 1 mM EDTA). After sonication and centrifugation, the supernatants were loaded onto a HisTrap HP column using an AKTA Prime Plus (GE healthcare, Chicago, IL, USA), which had been pre-equilibrated with a lysis buffer. The recombinant *Ha*HSL protein was eluted with an imidazole gradient from 50 to 300 mM. After extensive washing, the collected fractions were desalted with a storage buffer (20 mM Tris-HCl pH 8.5, 1 mM EDTA). The protein concentration was determined using a Biorad Protein assay kit (Bio-rad, Hercules, CA, USA) and the protein purity was confirmed using sodium dodecyl sulfate-polyacrylamide gel electrophoresis. The overall procedure yielded ≈5 mg of enzyme from 1 L of culture and the purified *Ha*HSL was stored at −20 °C. Site-directed mutagenesis of *Ha*HSL was conducted using the Quik-change site-directed mutagenesis method (Stratagene, La Jolla, CA, USA). All mutants (W23A, Y74A, F78A, W83A, S138A, and F245A) of *Ha*HSL were purified using the same method as described for wild-type *Ha*HSL.

### 2.3. Biochemical Assays

Activity staining was performed using native-PAGE incubated with Coomassie Brilliant Blue R-250 and 4-methylumbelliferone (4-MU) acetate [17]. For gel filtration analysis, purified *Ha*HSL was applied to a HiPrep Sephacryl S-200R column at a flow rate of 0.5 mL·min^−1^. Molecular weight determination was carried out using a Voyager BioSpectrometry system (NICEM, Seoul, Korea). Substrate specificities of *Ha*HSL and its mutants were analyzed using *p*-nitrophenyl (*p*-NP) esters and naphthyl esters. Specifically, the amounts of *p*-nitrophenol released from *p*-nitrophenyl esters with different chain lengths were measured at 405 nm using *p*-nitrophenyl acetate (*p-*NA), *p*-nitrophenyl butyrate (*p-*NB), *p*-nitrophenyl hexanoate (*p-*NH), *p*-nitrophenyl octanoate (*p-*NO), *p*-nitrophenyl decanoate (*p-*ND), *p*-nitrophenyl dodecanoate (*p-*NDD), and *p*-nitrophenyl phosphate (*p-*NPP). For naphthyl esters, the formation of naphthol was monitored at 315 nm using 1-naphthyl acetate (1-NA), 2-naphthyl acetate (2-NA), and 1-naphthyl butyrate (1-NB). All spectroscopic analyses were carried out in 20 mM Tris-HCl and 100 mM NaCl at pH 7.4 using an Epoch 2 Microplate Spectrophotometer (BioTek, Winooski, VT, USA). The standard assay solution included a 50 μM substrate in 20 mM Tris-HCl (pH 8.0) with 1 µg of *Ha*HSL, and the assay was run for 5 min at 25 °C.

The thermostability and pH stability of *Ha*HSL were investigated at different temperatures ranging from 20 to 60 °C and across a pH range from 3.0 to 10.0. CD (Circular dichroism) spectra and intrinsic fluorescence spectra were recorded using a J-715 spectropolarimeter (Jasco, Easton, MD, USA), and an FP-8200 spectrofluorometer (Jasco, Easton, MD, USA), respectively. Thermal unfolding of *Ha*HSL was monitored using the CD signals at 222 nm over a temperature range from 25 to 70 °C, which was controlled using a thermostatic cell holder. For intrinsic fluorescence spectra, the emission spectra from 300 to 400 nm were measured after excitation at 295 nm. The effects of chemicals (urea (from 0 to 5 M), NaCl (from 0 to 4M), glycerol (from 0 to 40%), Triton X-100 (from 0 to 20%), and Tween 20 (from 0 to 20%)) on the activity of *Ha*HSL were investigated after 1 h of incubation using *p*-nitrophenyl acetate (*p*-NA) as a substrate, and the enzyme activity of *Ha*HSL in buffer alone was defined as 100%.

Hydrolysis of 4-MU acetate or phosphate was also measured in an Eppendorf tube containing *Ha*HSL in a UV illumination box [16,17]. Freeze–thaw experiments were carried out with 20 cycles of freeze (−70 °C for 1 h) and thaw (25 °C for 1 h). In each cycle, the remaining enzyme activity of *Ha*HSL was determined using standard assay conditions.

For pH indicator-based colorimetric assays, *Ha*HSL was added to a phenol red-containing substrate solution. The substrates included lipids (glyceryl tributyrate, glyceryl trioleate, olive oil, and fish oil), tertiary alcohol esters (tertiary butyl acetate, α-terpinyl acetate, and linalyl acetate), and enantiomeric substrates (methyl (*R*)-(-)-3-hydroxyl-2-methylpropionate, methyl (*S*)-(-)-3-hydroxyl-2-methylpropionate, (1*R*)-(-)-menthyl acetate, and (1*R*)-(-)-menthyl acetate) [18]. Kinetic analyses of *Ha*HSL were carried out using various concentrations of *p-*nitrophenyl acetate (*p*-NA), *p-*nitrophenyl butyrate (*p*-NB), and *p-*nitrophenyl hexanoate (*p*-NH) as substrates, which were fit into the Michaelis–Menten equation. The maximum velocity (Vmax), turnover number (*k_cat_*), Michaelis–Menten constant (*K*_M_), and catalytic efficiency (*k_cat_/K*_M_) were all calculated from Lineweaver–Burk double reciprocal plots using GraphPad Prism 6.01 (GraphPad Softwar, San Diego, CA, USA). The amount of acetic acid released from the hydrolysis of the various substrates (tertiary alcohol esters (*tert*-butyl acetate, α-terpinyl acetate, and linalyl acetate), lipids (glyceryl tributyrate, glyceryl trioleate, olive oil, and fish oil), acetylated carbohydrates (α-D-mannose pentaacetate and β-D-galactose pentaacetate), and propionic acids (*rac*-naproxol acetate and (*S*)-naproxol acetate)) was quantitatively determined using an acetic acid kit (K-ACET, Acetyl-CoA Synthetase format, Megazyme, Chicago, IL, USA) according to the manufacturer’s guidelines.

### 2.4. Immobilization Methods

For the preparation of cross-linked enzyme aggregates (CLEA), 0.5 mg·mL^−1^ of *Ha*HSL was coprecipitated with 60% ammonium sulfate with glutaraldehyde, incubated overnight, and centrifuged [19]. The pellet (CLEA-*Ha*HSL) was resuspended and washed repeatedly until no enzyme activity was observed in the supernatant. The addition of *L*-Arg and magnetic Fe_3_O_4_ nanoparticles for the preparation of *L*-Arg-CLEA-*Ha*HSL and mCLEA-*Ha*HSL, respectively, were carried out as described previously [19,20]. For the preparation of an enzyme-inorganic hybrid nanoflower (hNF), 0.5 mg·mL^−1^ of *Ha*HSL was added to a 800 μM Cu^2+^ solution. The resulting mixtures were incubated, centrifuged, and washed repeatedly. The final pellets were re-suspended for further use. Scanning electron microscope (SEM) images were obtained at various magnifications (50,000×–100,000×) using a Carl Zeiss SUPRA 55VP (NICEM, Seoul, Korea). For the recycling experiments, three forms of immobilized *Ha*HSL (CLEA-*Ha*HSL, L-Arg-CLEA-*Ha*HSL, and mCLEA-*Ha*HSL) were reused after extensive washes in subsequent cycles.

### 2.5. Bioinformatic Analysis

The primary sequence of *Ha*HSL and related lipases were retrieved from the NCBI database using PSI-BLAST. Multiple sequence alignments were carried out using Clustal Omega [21] and ESPript [22]. A structural model of *Ha*HSL was generated using the structure of a putative esterase/lipase/thioesterase from *Ruegeria sp.* TM1040 (identity 57%, PDB code: 2PBL) as a template on the SWISS-MODEL server, which showed the highest sequence identity in the PDB. PyMOL was used for graphical representations of the *Ha*HSL structure.

## 3. Results and Discussion

### 3.1. Bioinformatic Analysis of HaHSL

In the chromosome of *H. arcticus*, an open reading frame encoding a novel hormone sensitive lipase (*Ha*HSL, locus tag: WP_039001964, 789 bp) was detected using in silico analysis. Sequence analysis showed that the molecular mass of *Ha*HSL was approximately 29.4 kDa, which consisted of a single polypeptide chain of 293 amino acids. No signal peptide sites or attributes of secretion were detected. The deduced protein sequence of *Ha*HSL shared the highest identity with α/β hydrolase from *Pseudoruegeria aquimaris* (68% identity, SLN30214), followed by α/β hydrolase from *Rhodobacteraceae bacterium* BAR1 (62% identity, AXX99873), α/β hydrolase from *Silicimonas algicola* (60% identity, AZQ68560), and α/β hydrolase from *Boseongicola aestuarii* (59% identity, SMX22590). However, none of these polypeptides were functionally or structurally characterized.

**Figure 1:**
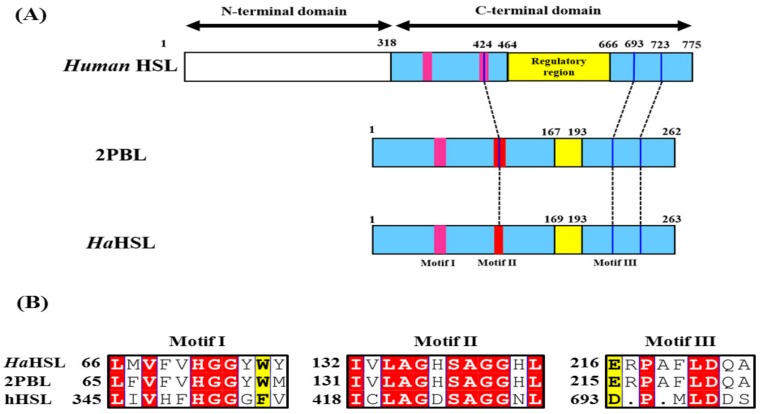
Structural organization of *Ha*HSL. (**A**) Schematic representations of human HSL (hHSL), 2PBL, and *Ha*HSL are shown. Three highly conserved sequence motifs are shown as red boxes and three catalytic amino acids are shown as black lines. Regulatory regions of these hormone-sensitive lipases are shown in yellow. (**B**) Sequence alignment of three conserved motifs (motif I, II, and III) in human HSL, 2PBL, and *Ha*HSL. Highly conserved residues are highlighted in red.

As shown in Figure 1A, *Ha*HSL, like other bacterial HSL family members, is homologous with the catalytic domain of human HSL. Based on sequence alignments, three highly conserved sequence motifs were identified among HSL family members (Figure 1B). Three amino acids of Ser^138^, Glu^216^, and His^244^ formed the catalytic triad, with Ser^138^ being located in a highly conserved GxSAGGxL motif. Furthermore, a highly conserved HGG motif, which contributed to the formation of an oxyanion hole for efficient catalysis, was identified in about 70 amino acids upstream of Ser^138^. In *Ha*HSL, Glu^216^ followed by His^244^ is located in the catalytic triad, although Asp was observed in the sequence of 2PBL or hHSL. In the sequence analysis, *Ha*HSL had high proportions of small amino acids, such as Ala (13.3%) and Gly (6.5%). Moreover, the proportion of acidic amino acids (Asp + Glu) was 14.9 and that of basic amino acids (Lys + Arg) was 10.7, which is frequently observed in halophilic enzymes [23,24].

### 3.2. Characterizations of HaHSL

**Figure 2:**
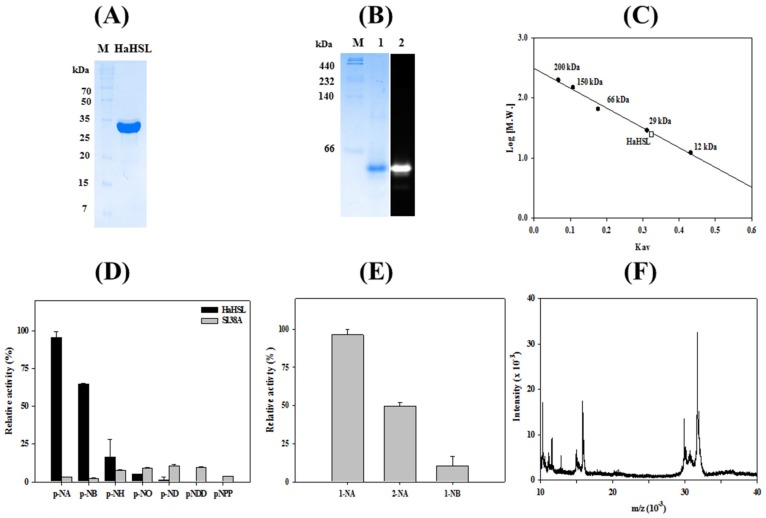
Biochemical properties of *Ha*HSL. (**A**) SDS–PAGE analysis. (**B**) Native-PAGE and activity staining analysis. (**C**) Gel filtration analysis of *Ha*HSL. (**D**) Substrate specificity of *Ha*HSL and its S138A mutant using *p*-nitrophenyl (*p*-NP) esters with different acyl chain lengths. (**E**) Activity of *Ha*HSL toward naphthyl derivatives. (**F**) Mass analysis of *Ha*HSL. Experiments of (**D**,**E**) were performed at least in triplicate.

Recombinant *Ha*HSL protein was highly purified using an immobilized metal-affinity column. The purified *Ha*HSL migrated as a single band of approximately ≈30 kDa using SDS-PAGE (Figure 2A). The activity of purified *Ha*HSL was initially analyzed via activity staining using 4-MU acetate. As shown in Figure 2B, a strong fluorescence was detected at the position where purified *Ha*HSL was located. In gel filtration chromatography, *Ha*HSL was eluted as a monomer (Figure 2C), which has different form than other recently reported bacterial HSL family lipases [10,25,26]. The hydrolytic activity of *Ha*HSL was analyzed using *p*-nitrophenyl (*p*-NP) esters with different fatty acid chain lengths. As shown in Figure 2D, *Ha*HSL had a strong substrate preference for *p*-NA (C_2_) or *p*-NB (C_4_), while the mutation of Ser^138^ abolished the hydrolytic activity of this enzyme for *p*-NP esters. In addition, almost no enzyme activity was observed for long-chain substrates of *p*-NO (C_8_), *p*-ND (C_10_), or *p*-nitrophenyl phosphate (*p*-NP). The preference for short-chain *p*-nitrophenyl esters was also observed in other members of bacterial HSL family members, such as E25 [25] or Est22 [26]. For naphthyl derivatives as substrates, the highest activity of *Ha*HSL was observed with 1-naphthyl acetate (1-NA), followed by 2-naphthyl acetate (2-NA) (Figure 2E). *Ha*HSL showed regioselectivity, which showed that activity toward 2-NA was only 48% of that toward 1-NA, respectively. Mass spectrometric analysis of *Ha*HSL showed a major [M + H]^+^ peak at *m*/*z* of 31,740 (Figure 2F).

**Figure 3:**
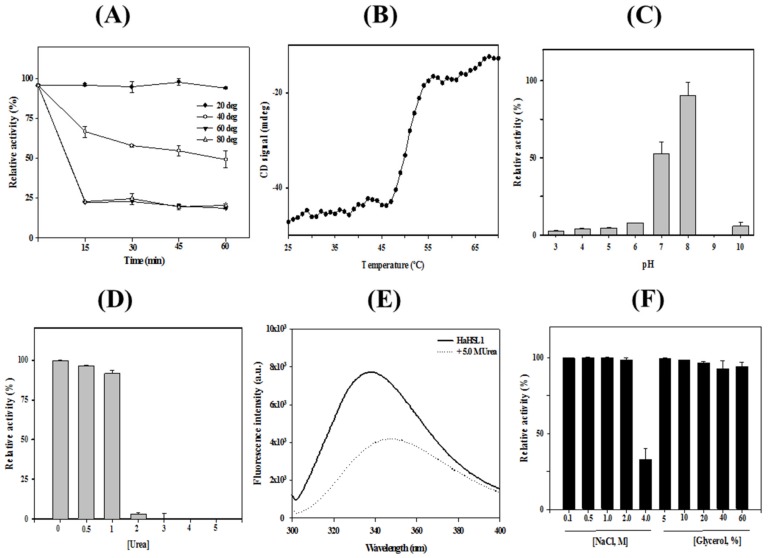
Stability of *Ha*HSL. (**A**) Thermal stability of *Ha*HSL was investigated at 20 °C, 40 °C, 60 °C, and 80 °C for 1 h. (**B**) Thermal unfolding of *Ha*HSL was monitored using CD analysis at 222 nm. (**C**) pH stability of *Ha*HSL was investigated from pH 3.0 to pH 8.0. (**D**) Activity of *Ha*HSL in the different concentrations of urea. (**E**) Urea-induced unfolding of *Ha*HSL was investigated using intrinsic fluorescence spectra. (**F**) Activity of *Ha*HSL in the presence of various concentrations of NaCl and glycerol. All experiments were performed at least in triplicate.

Thermostability of *Ha*HSL was investigated from 20 °C to 80 °C (Figure 3A). At 20 °C, almost 100% of the original activity was maintained after 1 h of incubation. However, at 40 °C, *Ha*HSL lost its activity gradually and retained only ≈65% of its initial activity after 30 min. At 60 °C, more than 80% of the enzyme activity was lost after 15 min. Thermal denaturation of *Ha*HSL was also monitored using a far-UV CD spectrum at 222 nm from 25 to 70 °C. In accordance with enzyme activity assays, *Ha*HSL showed very little change up to 48 °C, and the melting temperature was determined as being 52.8 °C (Figure 3B). *Ha*HSL displayed its maximal activity at pH 8.0, whereas ≈60% of this maximal activity was observed at pH 7.0 (Figure 3C).

Chemical stability against urea was investigated by measuring the activity of *Ha*HSL with increasing concentrations of urea. At 1.0 M urea, more than 95% of the initial activity was retained. However, at high concentrations over 2.0 M urea, little activity of *Ha*HSL was detected (Figure 3D). The chemical stability of *Ha*HSL was also investigated by monitoring the intrinsic fluorescence. In the native state, *Ha*HSL exhibited a λ_max_ at 334 nm, implying that all tryptophan residues of *Ha*HSL were located in the hydrophobic interior. However, a red shift of λ_max_ to 347 nm was observed with a remarkable reduction of fluorescence intensity with 5 M urea, suggesting that all tryptophan residues were mostly exposed to the solvent (Figure 3E). *Ha*HSL was also shown to be highly stable in the presence of high concentrations of NaCl and glycerol. Interestingly, unlike most halophilic enzymes, *Ha*HSL was highly active in the absence of NaCl. Furthermore, *Ha*HSL activity was shown to have ≈100% of its initial activity in the presence of 0.10–2.0 M NaCl (Figure 3F). Moreover, *Ha*HSL retained ≈35% of its original activity in 4.0 M NaCl. In addition, *Ha*HSL exhibited a strong tolerance to glycerol with almost no activity loss after incubation to the 60% glycerol condition.

**Figure 4:**
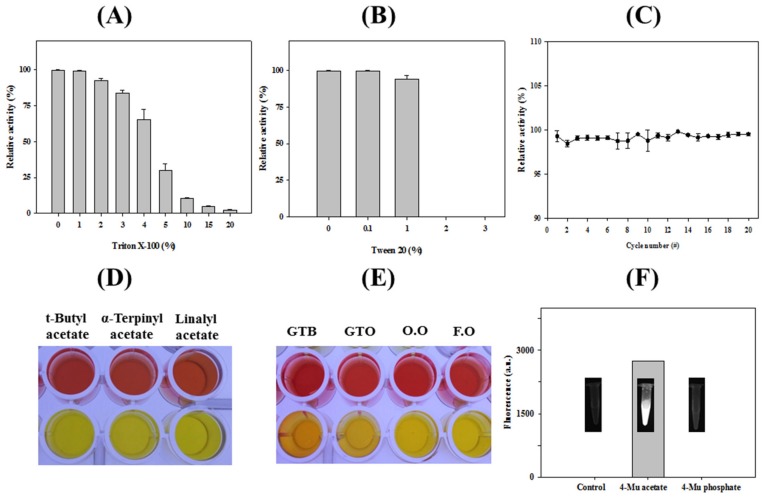
Enzymatic properties of *Ha*HSL. Stability of *Ha*HSL toward detergents was studied using various concentrations of (**A**) Triton X-100 and (**B)** Tween 20. (**C**) Freezing–thaw experiments of *Ha*HSL for 20 cycles. (**D**,**E**) A pH shift assay was performed for glyceryl esters (glyceryl tributyrate (GTB) and glyceryl trioleate (GTO)), and oils (olive oil (O.O.) and fish oil (F.O.)). (**F**) Fluorescence intensity due to the hydrolysis of 4-methylumbelliferyl (4 MU)-acetate and -phosphate by *Ha*HSL.

Effects of non-ionic detergents, such as Triton X-100 and Tween 20, on the activity of *Ha*HSL were investigated (Figure 4A,B). Specifically, *Ha*HSL generally retained over 30% of its activity with a 3% and 1% addition of Triton X-100 and Tween 20, respectively. This property could be attributed to the prevention of enzyme aggregation with increased substrate accessibility, which would be useful for industrial applications. On the contrary, the addition of 0.1% sodium dodecyl sulfate (SDS) resulted in a complete loss of *Ha*HSL activity (data not shown). The cold-stable property of *Ha*HSL were investigated using freezing–thaw cycles. As shown in Figure 4C, most of the initial activity was maintained after the 20th cycle, which showed that *Ha*HSL was highly stable at cold temperatures. The hydrolytic properties of *Ha*HSL for several lipids and tertiary alcohol esters were studied using a pH-indicator-based colorimetric assay [18,19,20]. As shown in Figure 4D, *Ha*HSL effectively hydrolyzed bulky tertiary acetyl esters, such as *tert*-butyl acetate, linalyl acetate, and α-terpinyl acetate. In addition, significant hydrolytic activity of *Ha*HSL for glyceryl tributyrate or glyceryl trioleate was detected based on the yellow color of the solution (Figure 4E). A high level of enzyme activity was also observed toward fish oil and olive oil. Furthermore, strong fluorescence from 4-methylumbelliferone was observed with 4-methylumbelliferyl acetate (4-MU acetate) and *Ha*HSL (Figure 4F). In addition, quantitative analysis was made using an acetic acid release assay with these substrates (Table 1). Interestingly, *Ha*HSL displayed a high activity for galactose pentaacetate, although no significant enzyme activity was observed for mannose pentaacetate. This promiscuous property of *Ha*HSL could be useful for industrial applications as a biocatalyst.

**Figure 5:**
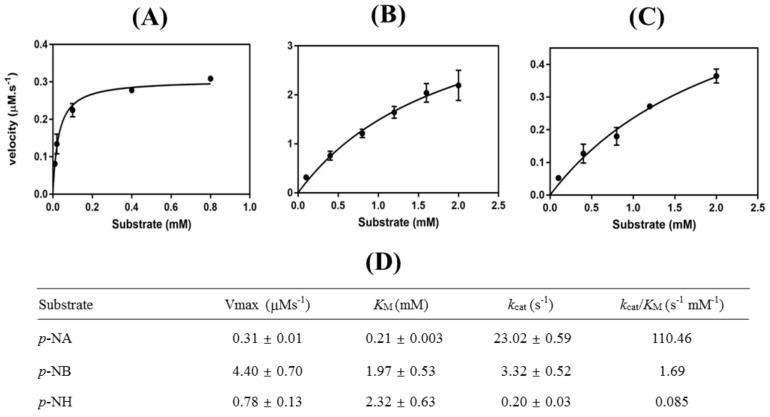
Kinetic analysis of *Ha*HSL. With different substrates of (**A**) *p*-NA, (**B**) *p*-NB, and (**C)** *p*-NH. Kinetic parameters of *Ha*HSL (**D)** were determined from Lineweaver–Burk plots [27]. All experiments were performed at least in triplicate.

The kinetic parameters of *Ha*HSL were determined using Michaelis–Menten kinetics by considering various concentrations of three different substrates (*p*-NA, *p*-NB, and *p*-NH). The maximum velocity (Vmax), Michaelis–Menten constant (*K_M_*), turnover number (*k_cat_*), and catalytic efficiency (*k_cat_/K_m_*) are shown in Figure 5. *K_M_* and *k_cat_* were 0.21 mM and 23.0 s^−1^, respectively, with *p*-NA as a substrate. The highest value for catalytic efficiency (110.4 s^−1^∙mM^−1^) was for *p*-NA, which is consistent with the substrate specificity (see also Figure 3A). As shown in Figure 5, *kcat*/*K_M_* for *p*-NA was determined to be ≈65-fold (1.69 s^−1^∙mM^−1^) and ≈1300 fold (0.085 s^−1^∙mM^−1^) higher than for *p*-NB and *p*-NH, respectively.

### 3.3. Immobilization of HaHSL

**Figure 6:**
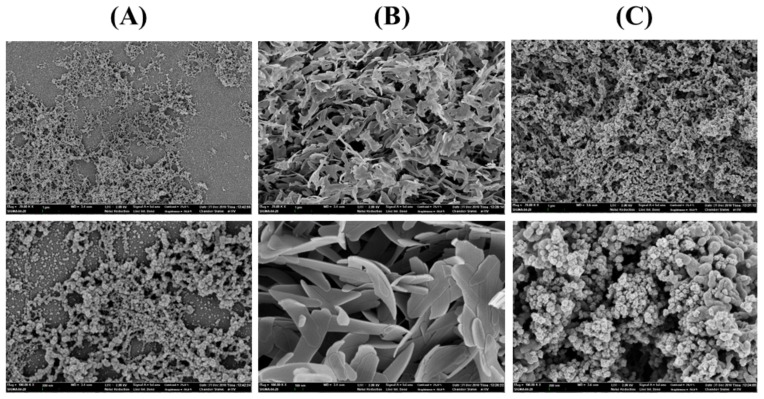
Immobilization of *Ha*HSL. Field emission scanning electron microscopic images of CLEA-Arg-*Ha*HSL (**A**), mCLEA-Arg-*Ha*HSL (**B**), and hNF-*Ha*HSL (**C**) were obtained at 50,000× (**upper**) and 150,000× (**lower**) magnifications.

Enzyme immobilization has been explored in a large variety of industrial applications. Cross-linked enzyme aggregates (CLEAs) is one of the highly studied methods to generate highly efficient immobilized biocatalysts [28]. To generate highly efficient immobilized biocatalysts, *Ha*HSL-CLEAs were prepared by precipitating *Ha*HSL with ammonium sulfate and glutaraldehyde. In our initial experiments, the immobilization efficiency of *Ha*HSL-CLEAs was relatively low compared to free *Ha*HSL (data not shown). Recently, it has been reported that the addition of Arg during CLEA formation could increase the stability of CLEAs [29]. As shown in Figure 6A, SEM images of *Ha*HSL-Arg-CLEAs showed that they were spherical and had an average particle size of ≈0.1–0.2 μm. The operational stability of *Ha*HSL-Arg-CLEAs was studied up to 15 cycles. After each cycle, *Ha*HSL-Arg-CLEAs were separated and washed for the next cycle. As shown in Figure 6A, *Ha*HSL-Arg-CLEAs were highly stable for four cycles, retaining ≈85% of original activity, while very little activity was detected in the fifth cycle.

In addition, organic-inorganic hybrid nanoflowers (hNFs) using several enzymes were reported to show enhanced activity and stability [30]. Herein, the formation and catalytic activity of hybrid nanoflowers containing *Ha*HSL was studied. Hybrid nanoflowers of *Ha*HSL were characterized as hierarchical peony-like structures assembled from interlaced nanoplates in SEM images (Figure 6B). The reusability studies were also carried out for ten cycles and the residual activity was over 80% after two successive cycles due to highly ordered and rigid structures of *Ha*HSL-hNFs. However, the catalytic activity of *Ha*HSL-hNFs exhibited a large drop in the third cycle.

**Figure 7:**
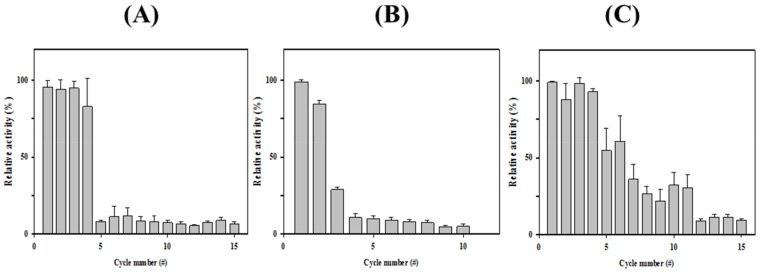
Reusabilities of immobilized *Ha*HSL. The reusability of CLEA-Arg-*Ha*HSL (**A**), mCLEA-Arg-*Ha*HSL (**B**), and hNF-*Ha*HSL (**C**) were investigated. All assays were performed at least in triplicate.

Furthermore, *Ha*HSL was immobilized as CLEAs on magnetite nanoparticles for an efficient separation process in industrial applications. Magnetic Fe_3_O_4_ nanoparticles were formulated and diffused in the reaction mixture, which could be selectively obtained using magnets [19]. To obtain magnetic CLEA of *Ha*HSL (mCLEA-*Ha*HSL), *Ha*HSL was coaggregated with nanoparticles, and then chemically cross-linked using glutaraldehyde. SEM images of *Ha*HSL-CLEAs confirmed the formation of globular structures with a diameter of ≈0.2–0.3 μm (Figure 6C). As shown in Figure 7, mCLEA-*Ha*HSL retained ≈65% of its original activity after the sixth cycle, which was 55% higher than *Ha*HSL-Arg-CLEAs. Similar behavior was also observed in magnetic CLEAs of various proteins [31]. Taken together, the immobilization of *Ha*HSL using three approaches (*Ha*HSL-Arg-CLEA, hNFs-*Ha*HSL, and mCLEA-*Ha*HSL) was effectively performed, which could be exploited for industrial applications of *Ha*HSL.

### 3.4. Effects of Mutations in the Substrate Binding Region

**Figure 8:**
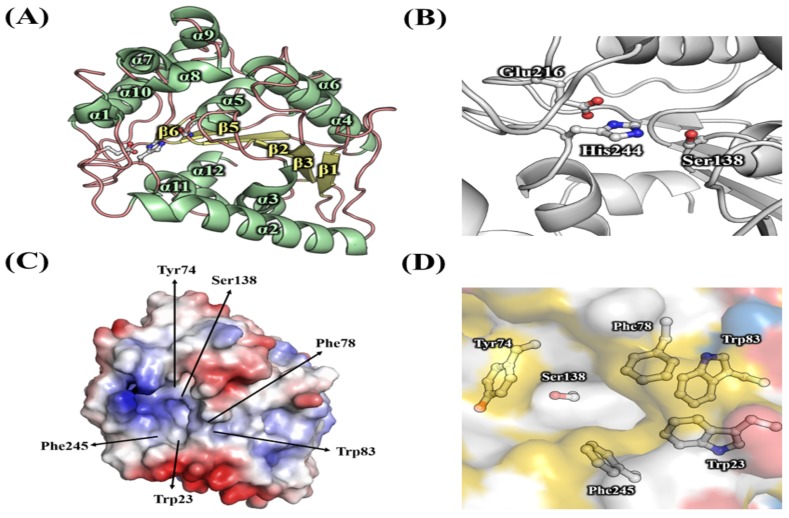
Structural model of *Ha*HSL. (**A**) The ribbon diagram of the *Ha*HSL structural model. (**B**) Catalytic amino acids of *Ha*HSL are shown in a ball-and-stick model. (**C**) Electrostatic representations of *Ha*HSL. The positions of five aromatic acids are shown in arrows. (**D**) The substrate-binding pocket of *Ha*HSL. Five aromatic amino acids are shown with a catalytic serine.

The structural model of *Ha*HSL was constructed based on a highly related crystal structure of a putative esterase/lipase/thioesterase (PDB code: 2PBL) from *Ruegeria sp.* TM1040. A molecular model of *Ha*HSL showed an α/β hydrolase fold with six large β-strands surrounded by ten α helices (Figure 8A). The putative catalytic triad of Ser^138^, Glu^216^, and His^244^ was positioned in close proximity on the surface for hydrolysis (Figure 8B). Interestingly, five aromatic amino acids were identified in the substrate-binding pocket of *Ha*HSL, which could control the size or shape of incoming substrates through hydrophobic interactions (Figure 8C). As shown in Figure 8D, the right side of the substrate binding pocket was surrounded by three amino acids of Phe^78^, Trp^83^, and Trp^23^, while Tyr^74^ and Phe^245^ were located in the left and bottom region, respectively.

**Figure 9:**
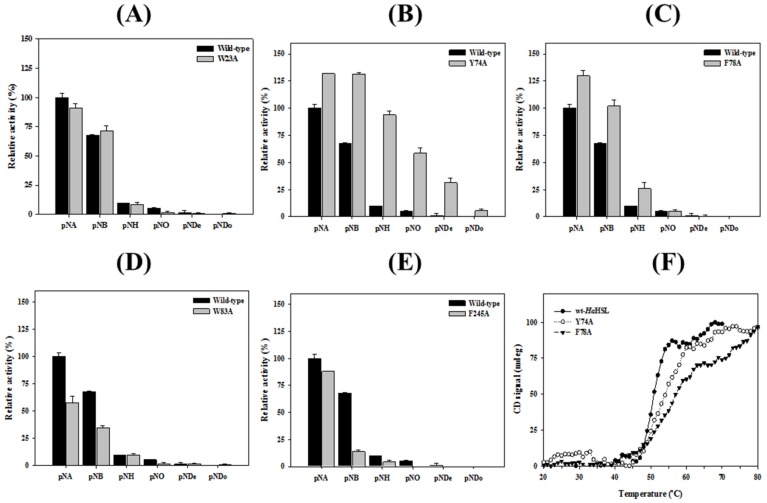
Substrate specificity of five aromatic acids mutants. Hydrolytic activities of *Ha*HSL and its five mutants toward different *p*-NP esters were measured. (**A**) W23A, (**B**) Y74A, (**C**) F78A, (**D**) W83A, and (**E**) F245A. (**F**) Thermal unfolding of *Ha*HSL and Y74A and F78A mutants were monitored using CD at 222 nm. All experiments were performed at least in triplicate.

To investigate the importance of these aromatic amino residues, five mutants (W23A, Y74A, F78A, W83A, and F245A) were prepared using site-directed mutagenesis, and the relative activity and substrate specificity of these mutants were studied. Compared with wild-type *Ha*HSL, the activity of W23A and F245A toward *p*-NA were slightly decreased. However, Y74A and F78A exhibited significantly enhanced activity, while the W83A mutant had a reduced level of hydrolytic activity (Figure 9). Similar behavior was also found toward 1-NA (data not shown). Specifically, Y74A and F78A retained ≈130% of the wild-type enzyme activity, while W83A and F245A showed only 65% of the wild-type activity. Interestingly, Y74A and F78A mutants showed substantial changes in substrate specificity, both of which could accept larger substrates, such as *p*-NB or *p*-NH (Figure 9B,C). This finding was more significant in Y74A, which showed a significantly higher level of activity toward *p*-NB compared with wild-type *Ha*HSL (Figure 9B). These results indicated that the side chains of aromatic residues in the substrate-binding pocket, especially in Tyr^74^ and Phe^78^ created a steric hindrance for controlling catalytic activity and substrate specificity in *Ha*HSL. Furthermore, the melting points of Y74A and F78A mutants were higher than that of wild-type *Ha*HSL (Figure 9F). Collectively, these results clearly suggested that these mutations in the substrate-binding region affected the catalytic activity, substrate specificity, and thermal stability of *Ha*HSL, which could be used to match specific needs for industrial applications.

**Figure 10:**
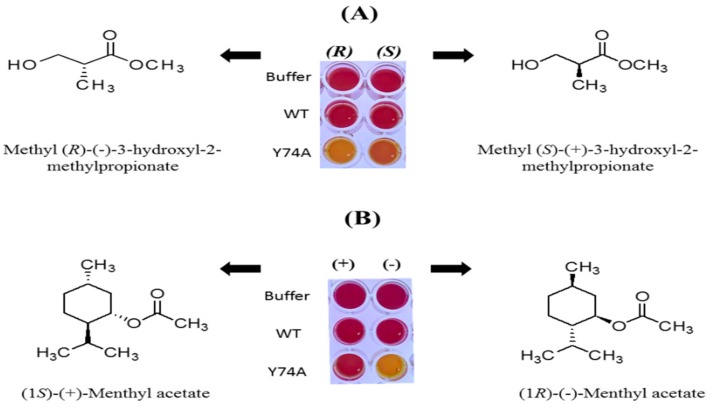
Enantioselectivity of wild-type *Ha*HSL and a Y74A mutant. (**A**) A pH shift assay toward the (*R*) or (*S*) form of methyl-3-hydroxyl-2-methylpropionate. (**B**) A pH shift assay toward the (*R*) or (*S*) form of menthyl acetate.

Using wild-type *Ha*HSL and a Y74A mutant, enantioselectivity toward two substrates was investigated using a pH-based colorimetric assay. As shown in Figure 10A, wild-type *Ha*HSL was shown to be very ineffective for the hydrolysis of these two enantiomeric substrates. However, the Y74A mutant was found to be highly active and selective for (*R*)-methyl-β-hydroxyisobutyrate. The solution containing the (*R*)-enantiomer turned became yellow to a greater degree, indicating the (*R*)-selectivity of this mutant. Similar enantioselectivity of the Y74A mutant was also observed in (1*R*)-(-)-menthyl acetate (Figure 10B). This is the first report that indicated a critical role of this residue in bacterial HSL family members. Considering the fact that enantiomeric behavior of lipases were of great importance in the preparation of chemical compounds in the field of pharmaceuticals and agrochemicals, this enantioselectivity of the Y74A mutant may help in understanding the mechanism of enantioselective hydrolysis of substrates [32].

## 4. Conclusions

Although bacterial HSL family members have attracted great interest owing to their industrial potential, no information was available on HSLs from the genus *Halocynthiibacter*. In summary, this work provides a molecular understanding of a novel cold-active *Ha*HSL for potential use in a large variety of biotechnological applications. Based on its unique property, such as high stability in detergents and a cold-active property, *Ha*HSL could be used as a molecular platform in protein engineering for the preparation of valuable biochemicals or drug intermediates [33,34]. The determination of the *Ha*HSL structure will allow for a direct comparison to other HSL family members, thus providing insights on the mechanism of action of *Ha*HSL. Structural studies of *Ha*HSL using X-ray crystallography are currently underway in our laboratory. In addition, molecular mechanisms underlying how mutations of aromatic acids may have improved the enzyme properties, such as strong stability, substrate promiscuity, and directed enantioselectivity, will be pursued in our future work to understand this enzyme at the molecular level.

## Figures and Tables

**Table 1:** Acetic acid release assay of *Ha*HSL toward various acetate compounds. The amount of acetic acid released from the hydrolysis of different acetate-containing substrates by *Ha*HSL were measured. The absorbance of acetic acid was detected at 340 nm. All experiments were performed at least in triplicate.

Substrates			Concentration	Acetic Acid (g/L)	
	**Tertiary alcohol esters**				
		*tert*-Butyl acetate	0.25 M	0.75 ± 0.0	
		α-Terpinyl acetate	0.25 M	0.58 ± 0.0	
		Linalyl acetate	0.25 M	0.54 ± 0.0	
	**Lipids**				
		Glyceryl tributyrate	1.0%	0.51 ± 0.0	
		Glyceryl trioleate	1.0%	0.64 ± 0.0	
		Olive oil	1.0%	0.68 ± 0.0	
		Fish oil	1.0%	0.70 ± 0.0	
	**Acetylated carbohydrates**				
		α-D-Mannose pentaacetate	10 mM	0.09 ± 0.0	
		β-D-Galactose pentaacetate	10 mM	0.91 ± 0.2	
	**Propionic acids**				
		(*Rac*)-Naproxol acetate	0.30 M	0.19 ± 0.0	
		(*S*)-Naproxol acetate	0.15 M	0.52 ± 0.0	
